# Comparison of Slow and Forced Vital Capacity on Ability to Evaluate Respiratory Function in Bulbar-Involved Amyotrophic Lateral Sclerosis

**DOI:** 10.3389/fneur.2022.938256

**Published:** 2022-06-28

**Authors:** Xin Huang, Chenfang Du, Qiong Yang, Dongsheng Fan

**Affiliations:** ^1^Department of Neurology, Peking University Third Hospital, Beijing, China; ^2^Beijing Key Laboratory of Biomarker and Translational Research in Neuro-Degenerative Diseases, Beijing, China; ^3^Key Laboratory for Neuroscience, National Health Commission/Ministry of Education, Peking University, Beijing, China

**Keywords:** amyotrophic lateral sclerosis, bulbar-involved, respiratory function tests, slow vital capacity, forced vital capacity, dysarthria

## Abstract

**Background and Objective:**

The percent-predicted forced vital capacity (FVC%) in the pulmonary function test (PFT) is generally used to evaluate the respiratory function in amyotrophic lateral sclerosis (ALS). The slow vital capacity (SVC) is another method to evaluate the respiratory function. Some neurologists found that the FVC% was not reflective of respiratory symptoms and the percent-predicted SVC (SVC%) was found to be higher in some patients with bulbar-onset ALS. We aimed to compare the percent predicted SVC (SVC%) with FVC% in evaluating the respiratory function and investigate the associations between the associations between clinical characteristics and the difference between the SVC% and the FVC% (SVC%-FVC%) in bulbar-involved ALS patients.

**Method:**

This prospective study included patients with bulbar-involved ALS who visited the Peking University Third Hospital between October 2020 and November 2021. They underwent comprehensive clinical assessments, including bulbar symptom assessments, revised ALS functional rating scale (ALSFRS-R), Rasch-Built Overall Amyotrophic Lateral Sclerosis Disability Scale (Roads), and PFTs. The group differences were analyzed using parametric and non-parametric tests.

**Results:**

A total of 59 participants were initially enrolled, and 51 of them were included in the final analysis. In patients with bulbar-involved ALS, the SVC% (73.82 ± 21.95%) was significantly higher (*p* = 0.013) than the FVC% (71.42 ± 23.15%). After controlling for other relevant variables, a partial correlation analysis showed a significant correlation (r = −0.352, *p* = 0.041) between ALSFRS-R1 score and SVC%-FVC%.

**Conclusion:**

Our prospective study found that the SVC% was significantly higher and more reflective of actual respiratory function than the FVC% in patients with bulbar-involved ALS. Furthermore, the severity of dysarthria was found to be positively correlated with SVC%-FVC%, providing a clinical marker for predicting SVC%-FVC%.

## Introduction

Amyotrophic lateral sclerosis is a progressive and fatal neurodegenerative disorder. The median survival time ranges from 2 to 5 years. Respiratory failure owing to the weakness of respiratory muscles is the leading cause of death in amyotrophic lateral sclerosis (ALS) (1). The American Thoracic Society/European Respiratory Society (ATS/ERS) recommends regular respiratory monitoring at baseline and every 3 months (2). The respiratory function can be assessed by several tests such as forced vital capacity (FVC), slow vital capacity (SVC), sniff nasal inspiratory pressure (SNIP), and maximal inspiratory pressure (MIP). However, novel indices such as SNIP and MIP have not been widely applied in clinic currently. FVC and SVC are still the most common approaches to evaluate the respiratory function in ALS. In addition, many patients could only measure the FVC and the SVC at home during the COVID-19 pandemic.

In clinical practice, some neurologists have found that FVC did not match the respiratory symptoms in some patients with bulbar-involved ALS. In such patients, pulmonary function tests (PFTs) suggested abnormal FVC, but these patients either did not have clinical symptoms of respiratory insufficiency or had only mild symptoms that were not consistent with the FVC. However, the SVC of these patients were observed to be higher. There were few previous studies on the difference between the FVC and the SVC in ALS. The studies of Andrews et al. (3) and Calvo, et al. (4) separately compared the FVC and the SVC on respiratory function assessments and role of predicting survival. Both studies showed that the FVC and the SVC were strongly correlated in ALS and less correlated in ALS with bulbar onset. Patients whose PFTs showed higher SVC than FVC were more likely to have bulbar symptoms. Thus, we speculated that the cause of the difference between the SVC and the FVC might be bulbar function impairment.

The SVC is another measurement in PFTs, consisting of a slow exhalation after a maximal inspiration (3). Previous studies have shown that the SVC and the FVC are interchangeable in respiratory assessments and survival prediction in ALS (3–6). However, in those with bulbar-involved ALS, we speculate that the SVC is theoretically less affected by impaired bulbar function since the SVC maneuver does not require the forced and fast exhalation needed for the FVC maneuver. Our study aimed to compare the percent-predicted SVC (SVC%) with the percent-predicted FVC (FVC%) in evaluating the respiratory muscle function in patients with bulbar-involved ALS. Additionally, we aimed to further investigate the type of patients in which it is appropriate to assess the respiratory function with SVC% rather than FVC% by exploring the associations between clinical features and the difference between the SVC% and the FVC% (SVC%-FVC%).

## Materials and Methods

### Study Design

**Figure 1:**
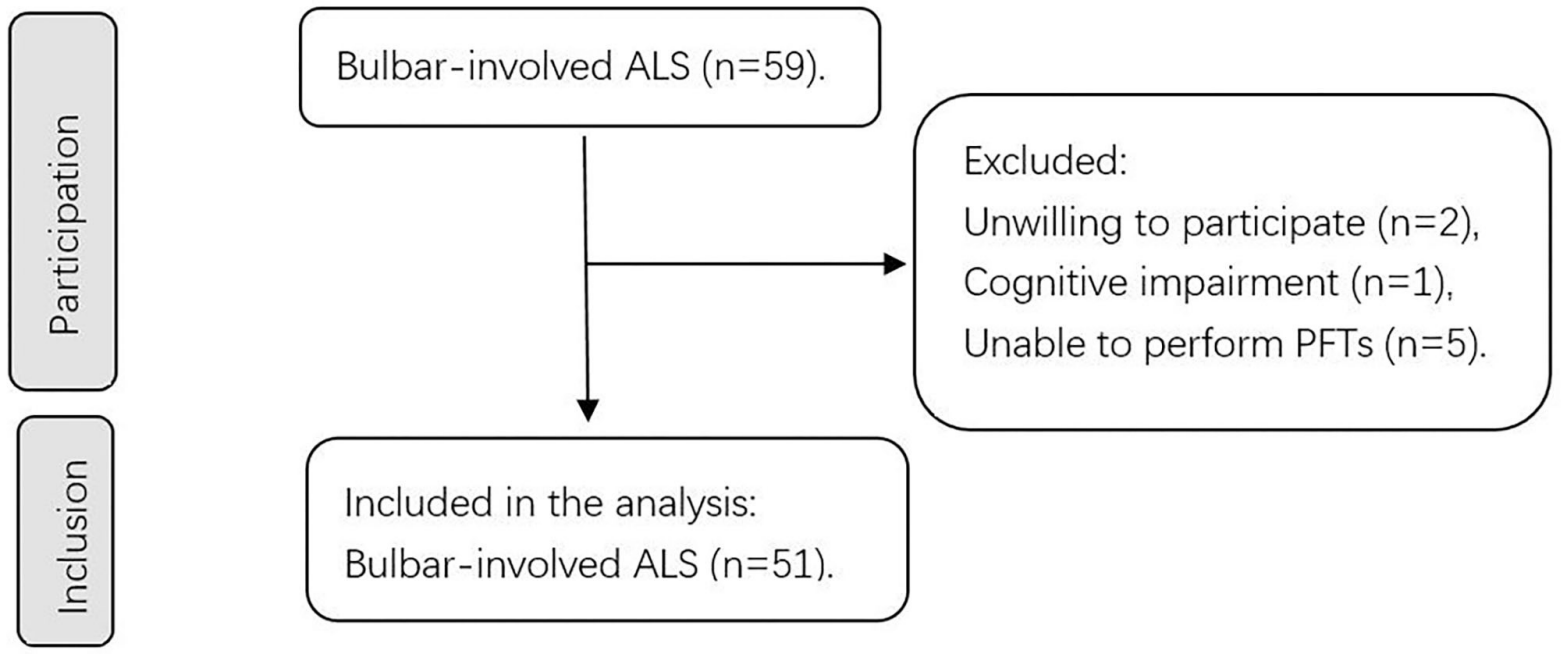
Flowchart of our study. A schematic summarizing the number of patients during participation and inclusion. ALS, amyotrophic lateral sclerosis; PFTs, pulmonary function tests.

This prospective study was conducted between 1 October 2020, and 30 November 2021, at the Peking University Third Hospital, Beijing, China. The study invited 59 patients with bulbar-involved ALS who fulfilled the revised El Escorial criteria for definite, probable, lab-supported probable and possible ALS (7). The exclusion criteria included (1) concomitant presence of severe complications (i.e., organ failure), (2) the inability to perform PFTs (i.e., after ophthalmologic surgeries; severe weakness of facial and oral muscles), (3) concomitant presence of respiratory diseases (i.e., chronic obstructive pulmonary disease and asthma), and (4) cognitive impairment or psychiatric disorders. Bulbar-involved ALS is defined as ALS with at least 1 clinical symptom indicating bulbar function impairment, including dysphagia, dysarthria, bucking, sialorrhea, and forced crying or laughter. At least 2 experienced doctors performed assessments for every participant. After assessing the eligibility based on the strict criteria listed above, 51 patients were finally included in the study. The institutional ethics committee of Peking University Third Hospital approved this study. Figure 1 shows a flow chart of the sample selection process in our study.

### Clinical Assessment

Detailed clinical features, including sex, age, body mass index (BMI), site of onset, age of onset, diagnostic delay, and diagnostic level, were collected. The Revised ALS Functional Rating Scale (ALSFRS-R) and the Rasch-Built Overall Amyotrophic Lateral Sclerosis Disability Scale (Roads) were used to evaluate the overall function (8–10). Each score related to bulbar function in the ALSFRS-R was noted separately in our study. Bulbar subscore of the ALSFRS-R (based on the first 3 questions of the ALSFRS-R) was noted. We used the King's College staging system (KCSS) to evaluate the staging of ALS (11, 12). The rate of disease progression (ΔFS) was calculated as follows: (48-[ALSFRS-R score] at time of diagnosis)/diagnostic delay (months).

### Pulmonary Function Tests

The PFTs were strictly performed in accordance with the ATS/ERS guidelines by the same 2 experienced technicians with the same type of standard volumetric spirometer (Jaeger spirometer, Germany) (13). All measurements were made in a sitting position with nose clips for nose occlusion. For the FVC maneuver, patients had to forcefully and quickly exhale after full inhalation to the total lung volume. For the SVC maneuver, maximal exhalation in a slow and gentle way was encouraged after full inhalation. At least two measures were taken to reduce loss in exhalation. First, we consulted several senior experts before beginning our study and chose an oblate port which was suitable for patients with lip weakness. Second, experts instructed the patients to use hands to help wrap the ports with lips. The best result from 3 measures was retained. The percent predicted (%) was calculated for analysis. We collected the FVC%, the SVC%, and their difference (SVC%-FVC%) for further analysis.

### Statistical Analysis

We used the SPSS 26.0 software (IBM SPSS Statistics, IBM Corporation) to analyze the data. The data are displayed as the means (standard deviations) for quantitative variables with a normal distribution and as the medians (interquartile range; IQR) for other variables. The data for categorical variables are shown as proportions (%). For variables with normally distributed data, the paired-sample *t* tests were used to compare the data between 2 groups. For variables with non-normally distributed data, the Mann–Whitney U tests were used to compare the data between 2 groups, and the Kruskal–Wallis tests were used to compare the data between more than 2 groups. Potentially relevant factors extracted from a univariate analysis (*p* < 0.10) were included in the partial correlation analysis model. The significance level was set at a 2-tailed *p* < 0.05.

In addition to evaluate SVC%-FVC% results in our study, we looked through other studies including normal people as well. Thus, we calculated the standardized mean difference (SMD), which was a value used to evaluate the similarity between 2 groups (14). The smaller the SMD is, the smaller the difference between these 2 groups.


$$\begin{array}{lll} SMD & = & \frac{{\bar{X}}_{1}\;-{\bar{X}}_{2}}{s_{p}}\\ sp & = & \sqrt{\frac{\left(n_{1}-1\right)s_{1}^{2}+\left(n_{2}-1\right)s_{2}^{2}}{\left(n_{1}-1\right)+\left(n_{2}-1\right)}} \end{array}$$

${\bar{x}}_{1}$ is the mean value of group 1 and ${\bar{x}}_{2}$ is the mean value of group 2. *s*_*p*_ is the pooled standard deviation, *n*_1_ and *n*_2_ are the sample sizes of the two groups. *s*_1_ is the standard deviation of group 1; and *s*_2_ is the standard deviation of group 2.

## Results

### Demographic and Clinical Information

Our study initially invited 59 participants, and 3 were excluded because of unwillingness and cognitive impairment. Moreover, 5 patients could not undergo PFTs, which resulted in 51 participants for inclusion and statistical analysis (Figure 1). These 5 patients were all bulbar-onset ALS and all started with dysarthria (basic information shown in Supplementary Material). Table 1 shows the demographic and clinical features of these 51 patients at baseline. The mean age was 55.5 (13.7) years old, and the mean BMI was 23.1 kg/m^2^. 28 of the 51 patients (54.9%) were male. Regarding the diagnostic level, 25 (49.0%) were clinically definite ALS, 19 (37.3%) were clinically probable ALS, 2 (3.9%) was lab-supported probable ALS, and 5 (9.8%) were clinically possible ALS. Regarding staging by the KCSS, 3 (5.9%) were in stage 1, 13 (25.5%) were in stage 2, 18 (35.3%) were in stage 3, and 17 (33.3%) were in stage 4. The mean age of onset was 53.2 (15) years old, and the median diagnostic delay was 12 (6–19) months. All participants were assessed with the ALSFRS-R. The median total score was 39 (35–41) and the median bulbar subscore was 10 (8–11). 44 of them were assessed with the Roads, and the mean score was 83.4 (12.9).

**Table 1:** Baseline characteristics of the patients examined in our study.

	**Bulbar-involved ALS, *n* = 51**
**Demographics**	
Age, mean (SD), y	55.5 (13.7)
Sex, no. (%), male	28 (54.9)
BMI, mean (SD), kg/m^2^	23.1 (3.6)
**Disease characteristics**	
Diagnosis level, no. (%)	
Definite	25 (49.0)
Probable	19 (37.3)
Lab-supported probable	2 (3.9)
Possible	5 (9.8)
KCSS, no. (%)	
Stage 1	3 (5.9)
Stage 2	13 (25.5)
Stage 3	18 (35.3)
Stage 4	17 (33.3)
Onset site, bulbar, no. (%)	22 (43.1)
Age of Onset, mean (SD), y	53.2 (15.0)
Diagnostic delay, median (IQR),months	12 (6–19)
ALSFRS-R, median (IQR)	39 (35–41)
B sub-score, median (IQR)	10 (8–11)
Roads score, mean (SD)^a.^	83.4 (12.9)

*ALS, amyotrophic lateral sclerosis; BMI, body mass index; KCSS, King's College staging system; ALSFRS-R, ALS functional rating scale-revised; B sub-score, bulbar sub-score of ALSFRS-R; Roads, Rasch-Built Overall Amyotrophic Lateral Sclerosis Disability Scale; SD, standard deviation; IQR, interquartile range*.

a *Forty-four of all 51 patients completed roads*.

### Difference Between SVC% and FVC%

**Figure 2:**
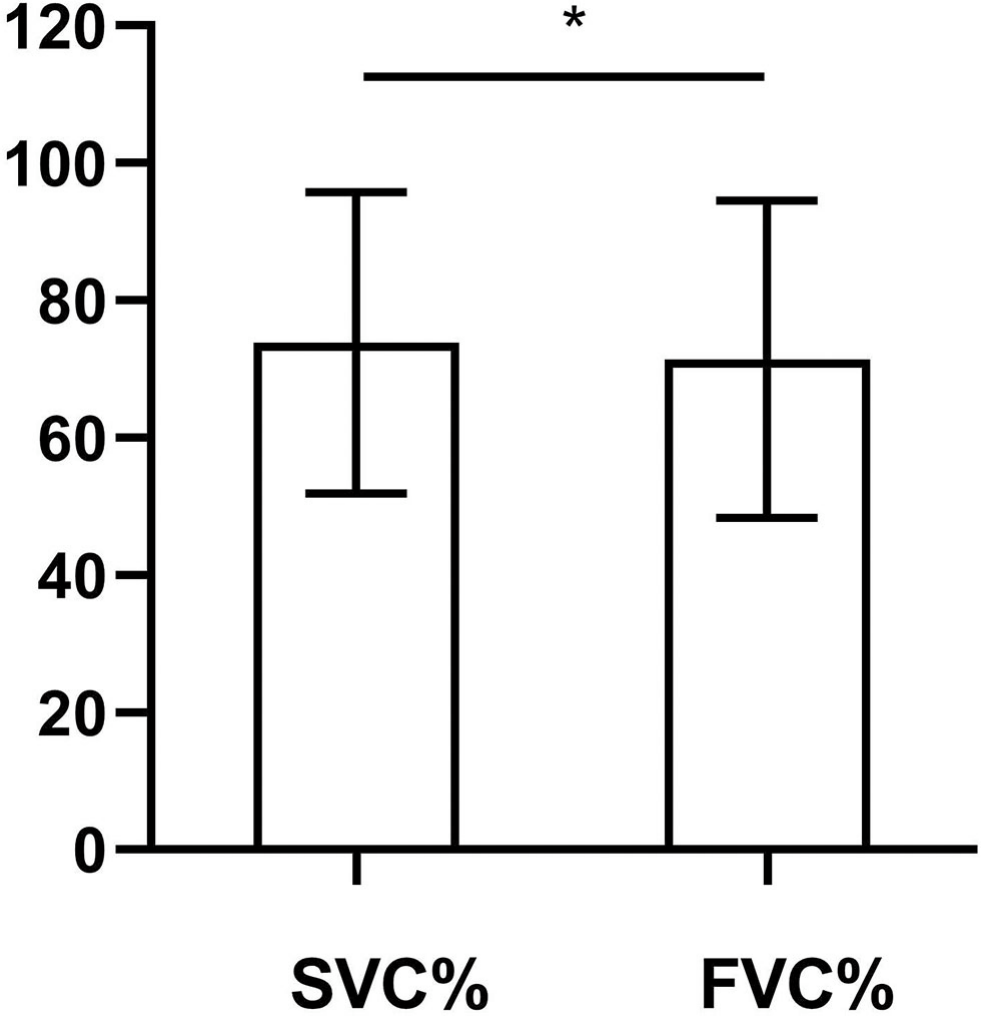
Comparison of SVC% and FVC% in bulbar-involved ALS (*n* = 51). SVC% (73.82 ± 21.95) was significantly higher than FVC% (71.42 ± 23.15). SMD = 0.51. *p* = 0.013. SVC%, percent predicted SVC values; FVC%, percent predicted FVC values; ALS, amyotrophic lateral sclerosis; SMD, standardized mean difference. *p < 0.05.

Figure 2 shows the difference between the SVC% and the FVC% in our study. The mean SVC% was 73.82% (21.95%), and the mean FVC% was 71.42% (23.15%). The SVC% was significantly higher (*p* = 0.013) than the FVC%. The SMD of our study was 0.51. In the analysis of the difference between the SVC% and the FVC% in patients with bulbar-involved ALS, we needed to consider the difference in normal people. We used the study of Saint-Pierre et al. (15), which involved 13,893 outpatients with total lung capacity at or above the lower limit of normal as a reference. The SMD in this reference study was calculated as 0.39. As a result, the SMD of our study, which represented the size of the difference between the SVC% and the FVC%, was larger than that of the reference study. This demonstrated that the difference between the SVC% and the FVC% in bulbar-involved ALS was significantly greater than that in normal people.

### Correlation of Clinical Data With SVC%-FVC%

To explore the appropriate kind of patients for assessing respiratory function with SVC%, we analyzed the correlation between clinical features and SVC%-FVC%. The participants were divided into 2 groups based on median SVC%-FVC%: one group included patients with SVC%-FVC% ≥ 1.00% (*n* = 28), and the other group included patients with SVC%-FVC% <1.00% (*n* = 23). Comparisons of demographic features, clinical features, bulbar function assessments by symptoms and scores are shown in Table 2. Due to the small sample size of this study, variables that were likely to be associated with SVC%-FVC% (*p* < 0.10) were included in a partial correlation analysis model. Thus, we included age (*p* = 0.074), KCSS (*p* = 0.008), ALSFRS-R1 (*p* = 0.01), ALSFRS-R3 (*p* = 0.022), ALSFRS-R (*p* = 0.047) scores, bulbar subscore of ALSFRS-R (*p* = 0.073), and Roads scores (*p* = 0.034) in the analysis model (Table 3). After controlling for other relevant variables, the partial correlation analysis showed a significant correlation (r = −0.352, *p* = 0.041) between ALSFRS-R1 score and SVC%-FVC%. A participant with a lower ALSFRS-R score, which indicates more severe dysarthria, is likely to have a higher SVC%-FVC% value. Thus, it is more appropriate for patients with severe dysarthria to assess respiratory function with SVC% than FVC%. No significant relationship was observed for the other variables.

**Table 2:** The difference of demographics, clinical characteristics and scale scores at baseline between 2 groups which were divided by median SVC%-FVC%.

**Variables**	**Group 1 (SVC%-FVC% <1.00%) (*n* = 23)**	**Group 2 (SVC%-FVC%≥1.00%) (*n* = 28)**	**p**
**Demographics**			
Age, mean (SD), y	51.74 (13.27)	58.61 (13.43)	0.074^*^
Sex, no. (%), male	15 (65.20)	13 (46.40)	0.183
BMI, mean (SD), kg/m^2^	22.92 (3.87)	23.26 (3.40)	0.744
**Clinical characteristics**			
Age of onset, mean (SD), y	49.96 (13.25)	55.79 (16.07)	0.170
Diagnostic delay, median (IQR),months	12 (5–24)	10 (6–14.75)	0.314
Onset site, no. (%), bulbar	8 (34.80%)	14 (50%)	0.277
KCSS, no. (%)			
1	1 (4.30)	2 (7.10)	0.008^*^
2	6 (26.10)	7 (25)	
3	13 (56.50)	5 (17.90)	
4	3 (13.00)	14 (50)	
ΔFS, median (IQR)	0.75 (0.29–1.90)	1.25 (0.77–1.44)	0.110
Baseline SVC%, mean (SD)	79.04 (19.11)	69.54 (23.51)	0.125
**Clinical symptoms**			
Forced crying or laughter, no. (%)	8 (34.80)	8 (28.60)	0.764
Dysphagia, no. (%)	11 (47.80)	18 (64.30)	0.269
Bucking, no. (%)	16 (69.60)	21 (75)	0.757
Sialorrhoea, no. (%)	15 (65.20)	16 (57.10)	0.580
Dysarthria, no. (%)	21 (91.30)	25 (89.30)	1.000
**Scale scores at baseline**			
ALSFRS-R, median (IQR)	40 (38–42)	37.50 (34–40)	0.047^*^
ALSFRS-R1 (speech), median (IQR)	3 (3–3)	2 (2–3)	0.010^*^
ALSFRS-R2 (salivate), median (IQR)	3 (3–4)	3 (3–4)	0.992
ALSFRS-R3 (swallow), median (IQR)	4 (3–4)	3 (3–4)	0.022^*^
B sub-score, median (IQR)	10 (9–11)	8.50 (8–10)	0.073^*^
Roads^a^, mean (SD)	87.30 (13.95)	79.14 (10.23)	0.034^*^

*SVC%, percent predicted SVC values; FVC%, percent predicted FVC values; BMI, body mass index; KCSS, King's College staging system; ΔFS, (48-ALSFRS-R at time of diagnosis)/diagnostic delay (months); ALSFRS-R, ALS functional rating scale-revised; B sub-score, bulbar sub-score of ALSFRS-R; Roads, Rasch-Built Overall Amyotrophic Lateral Sclerosis Disability Scale; SD, standard deviation; IQR, interquartile range*.

a *Forty-four of all 51 patients completed roads*.

* *p < 0.1*.

**Table 3:** Partial correlation analysis between characteristics and SVC%-FVC% of the patients with bulbar-involved ALS.

**Variables**	**r**	**p**
Age	0.057	0.750
KCSS	0.064	0.720
ALSFRS-R1 (speech)	−0.352	0.041^*^
ALSFRS-R3 (swallow)	−0.095	0.594
ALSFRS-R	−0.019	0.916
B sub-score	0.288	0.099
Roads	0.040	0.823

*SVC%, percent predicted SVC values; FVC%, percent predicted FVC values; KCSS, King's College staging system; ALSFRS-R, ALS functional rating scale-revised; B sub-score, Bulbar sub-score of ALSFRS-R; Roads, Rasch-Built Overall Amyotrophic Lateral Sclerosis Disability Scale*.

* *p < 0.05*.

## Discussion

Non-invasive ventilation is the standard therapy for respiratory insufficiency in ALS. The time of initiation mainly depends on the FVC% in PFTs (16). The FVC maneuver requires forced and fast exhalation, which is easily affected by other factors. For example, patients with bulbar and facial disturbances may not adequately perform FVC maneuvers, leading to lower FVC% compared with the actual ventilator function of these patients. Some patients with bulbar-involved ALS were observed to complain of no or mild respiratory symptoms with low FVC% (17). Hence, the FVC% was unable to precisely assess the respiratory muscle function in such patients. Given this situation, the appropriate timing for non-invasive ventilation (NIV) initiation could not be precisely determined by doctors. In addition, compliance with NIV was reported to be poor in bulbar-involved patients (18). For these 2 reasons, these patients were doubtful and found it difficult to adhere to treatment protocols given by doctors. We speculated that the SVC% might be less affected by bulbar and facial disturbances because of its smoother nature and therefore designed this study.

This study shows a significant difference between the SVC% and the FVC% in patients with bulbar-involved ALS. To indirectly demonstrate that the cause of the difference was impaired bulbar function, we referred to a previous study (15) that included individuals with normal respiratory function. The difference between the SVC% and the FVC% in our study was more significant than that in the reference study. Furthermore, a number of previous studies have shown that the SVC and the FVC are interchangeable in respiratory assessments and survival prediction in ALS (3–6). Therefore, we believe the difference found in our study is valuable and reflects impairments in bulbar function. Several previous studies showed that FVC may underestimate SVC in other diseases (15, 19–22). In bulbar-involved ALS, patients are required to exhale forcefully and quickly with the help of facial and bulbar muscles for the FVC maneuver. Expiratory imperfections caused by paralysis of glossopharyngeal muscles might lead to a lower FVC% in patients with bulbar-involved ALS. We speculated that the FVC% overestimated the loss of respiratory function in these patients, and the SVC% was able to reduce the misjudgment. Thus, we speculated that the SVC% was more reflective of the actual respiratory function than the FVC%.

To explore the type of patients in which the SVC% would more appropriately assess the respiratory function, we analyzed the correlation between SVC%-FVC% and the clinical features of patients, including their demographic features, disease-related features, and ALSFRS-R and Roads scores. Consequently, ALSFRS-R1 score was found to be significantly correlated with SVC%-FVC%. The ALSFRS-R1 question (scored from 0 to 4) is about speech assessment of ALS patients. A score of 4 indicates normal speech processes, 3 indicates detectable speech disturbance, 2 indicates intelligible speech with repeating, 1 indicates speech combined with non-vocal communication, and 0 indicates the loss of useful speech. We found that patients with more severe dysarthria had a higher SVC%-FVC% values. This suggested us that we should focus on the SVC% in patients with severe dysarthria to precisely assess their respiratory function. Furthermore, there were 5 patients who were unable to perform the FVC maneuver in our study. Arterial blood gas tests were performed, and none of these patients showed signs of respiratory failure. After clinical assessments and analysis, we hypothesized that the reasons were glossopharyngeal weakness and cough. We found that all of them were bulbar onset, and dysarthria was their first and main clinical manifestation. Unfortunately, we could not perform the SVC maneuver for these patients due to procedural problems. However, these observations partially supported the notion that the FVC% was unable to assess the respiratory function of ALS patients with dysarthria as their main symptom.

The reason why dysarthria was significantly related to SVC%-FVC% remains unclear. Speech is produced and regulated by nearly 70 highly coordinated muscles (23). Several mechanisms, such as pronunciation and resonance, cooperate with each other to ensure speech production (24). Dysarthria is caused by muscular paralysis of the face, tongue, palate, pharynx, and larynx (25). Weakness and reduced flexibility of these muscles, as well as disrupted antagonist synergy, can result in dysarthria in patients with bulbar-involved ALS (23). Forced and fast exhalation requires the coordinated work of all respiratory muscles, so we speculated that the mechanisms underlying dysarthria might be the reason for the high SVC%-FVC% in our study.

This study had several limitations. First, strict inclusion and exclusion criteria led to a small sample size, which may have partially affected the statistical power. Second, bulbar assessments were performed by traditional methods, such as symptom assessments and ALSFRS-R, Roads scores in our study. We expect future studies will use novel technologies such as automatic speech recognition technology for speech assessments (26) and oral manometry for swallowing assessments (27). Additionally, our study was conducted in a single center. Multicenter studies are needed to verify our findings.

## Conclusion

To conclude, our findings indicated that the SVC% was significantly higher and more reflective of actual respiratory function than the FVC% in patients with bulbar-involved ALS. The difference was significantly related to dysarthria. Thus, it is helpful for doctors to assess the respiratory function with SVC% in ALS patients with severe dysarthria. Further studies are still needed.

## Data Availability Statement

The raw data supporting the conclusions of this article will be made available by the authors, without undue reservation.

## Ethics Statement

Written informed consent was obtained from the individual(s) for the publication of any potentially identifiable images or data included in this article.

## Author Contributions

DF, QY, and XH conceived and designed the study. XH and CD conducted data management and statistical analysis. XH drafted the manuscript. DF and QY revised the manuscript. DF provided financial support. All authors contributed to the article and approved the submitted version.

## Funding

This study was funded by the National Natural Science Foundation of China (81873784 and 82071426) to DF and the Clinical Cohort Construction Program of Peking University Third Hospital (BYSYDL2019002 to DF).

## Conflict of Interest

The authors declare that the research was conducted in the absence of any commercial or financial relationships that could be construed as a potential conflict of interest.

## Publisher's Note

All claims expressed in this article are solely those of the authors and do not necessarily represent those of their affiliated organizations, or those of the publisher, the editors and the reviewers. Any product that may be evaluated in this article, or claim that may be made by its manufacturer, is not guaranteed or endorsed by the publisher.

## Abbreviations

ALS: Amyotrophic lateral sclerosis
PFT: Pulmonary function test, FVC Forced vital capacity
SVC: Slow vital capacity, FVC% Percent predicted forced vital capacity value
SVC%: Percent predicted slow vital capacity
SVC%-FVC%: The difference between SVC% and FVC%
ALSFRS-R: Revised ALS functional rating scale
Roads: Rasch-Built Overall Amyotrophic Lateral Sclerosis Disability Scale.

## References

[1] Dorst JA-O Ludolph AC. Non-invasive ventilation in amyotrophic lateral sclerosis. Ther Adv Neurol Disord. (2019) 2019:1756–2856. 10.1177/1756286419857040 31258624 PMC6589990

[2] Miller RG Jackson CE Kasarskis EJ England JD Forshew D Johnston W. Practice parameter update: the care of the patient with amyotrophic lateral sclerosis: drug, nutritional, and respiratory therapies (an evidence-based review): report of the quality standards subcommittee of the American academy of neurology. Neurology. (2009) 73:1218–26. 10.1212/WNL.0b013e3181bc0141 19822872 PMC2764727

[3] Andrews JA Meng L Kulke SF Rudnicki SA Wolff AA Bozik ME. Association between decline in slow vital capacity and respiratory insufficiency, use of assisted ventilation, tracheostomy, or death in patients with amyotrophic lateral sclerosis. JAMA Neurol. (2018) 75:58–64. 10.1001/jamaneurol.2017.3339 29181534 PMC5833488

[4] Calvo AA-O Vasta R Moglia C Matteoni E Canosa A Mattei A. Prognostic role of slow vital capacity in amyotrophic lateral sclerosis. J Neurol. (2020) 267:1615–21. 10.1007/s00415-020-09751-1 32052165

[5] Fernandez JA-O Castellano MA-O Vianna FA-OX Nacif SA-O Rodrigues RA-O Jr Rodrigues SA-O. Clinical and functional correlations of the difference between slow vital capacity and FVC. J Bras Pneumol. (2019) 46:e20180328. 10.1590/1806-3713/e20180328 31859814 PMC7462666

[6] Pinto S de Carvalho M. Correlation between forced vital capacity and slow vital capacity for the assessment of respiratory involvement in amyotrophic lateral sclerosis: a prospective study. Amyotroph Lateral Scler Frontotemporal Degener. (2017) 18:86–91. 10.1080/21678421.2016.1249486 27915482

[7] Brooks BR Miller RG Swash M Munsat TL. El Escorial revisited: revised criteria for the diagnosis of amyotrophic lateral sclerosis. Amyotroph Lateral Scler Other Motor Neuron Disord. (2000) 1:293–9. 10.1080/146608200300079536 11464847

[8] Cedarbaum JM Stambler N Malta E Fuller C Hilt D Thurmond B. The ALSFRS-R: a revised ALS functional rating scale that incorporates assessments of respiratory function. BDNF ALS study group (phase III). J Neurol Sci. (1999) 169:13–21. 10.1016/s0022-510x(99)00210-5 10540002

[9] Fournier CN Bedlack R Quinn C Russell J Beckwith D Kaminski KH. Development and validation of the rasch-built overall amyotrophic lateral sclerosis disability scale (ROADS). JAMA Neurol. (2020) 77:480–8. 10.1001/jamaneurol.2019.4490 31886839 PMC6990811

[10] Sun C Fournier CN Ye S Zhang N Ma Y Fan DA-O. Chinese validation of the rasch-built overall amyotrophic lateral sclerosis disability scale. Eur J Neurol. (2021) 28:1876–83. 10.1111/ene.14811 33686758

[11] Luna JA-O Couratier P Lahmadi S Lautrette G Fontana AA-O Tortelli R. Comparison of the ability of the King's and MiToS staging systems to predict disease progression and survival in amyotrophic lateral sclerosis. Amyotroph Lateral Scler Frontotemporal Degener. (2021) 22:478–85. 10.1080/21678421.2021.1903506 33829938

[12] Roche JC Rojas-Garcia R Scott KM Scotton W Ellis CE Burman R. A proposed staging system for amyotrophic lateral sclerosis. Brain. (2012) 135:847–52. 10.1093/brain/awr351 22271664 PMC3286327

[13] Graham BL Steenbruggen I Miller MR Barjaktarevic IZ Cooper BG Hall GL. Standardization of spirometry 2019 update. An official American thoracic society and european respiratory society technical statement. Am J Respir Crit Care Med. (2019) 200:e70–88. 10.1164/rccm.201908-1590ST 31613151 PMC6794117

[14] Andrade C. Mean difference, standardized mean difference (SMD), and their use in meta-analysis: as simple as it gets. J Clin Psychiatry. (2020) 81:20f13681. 10.4088/JCP.20f13681 32965803

[15] Saint-Pierre M Ladha J Berton DC Reimao G Castelli G Marillier M. Is the slow vital capacity clinically useful to uncover airflow limitation in subjects with preserved FEV(1)/FVC ratio? Chest. (2019) 156:497–506. 10.1016/j.chest.2019.02.001 30768928

[16] Shoesmith C Abrahao A Benstead T Chum M Dupre N Izenberg A. Canadian best practice recommendations for the management of amyotrophic lateral sclerosis. CMAJ. (2020) 192:E1453–68. 10.1503/cmaj.191721 33199452 PMC7683000

[17] Singh D Verma R Garg RK Singh MK Shukla R Verma SK. Assessment of respiratory functions by spirometry and phrenic nerve studies in patients of amyotrophic lateral sclerosis. J Neurol Sci. (2011) 306:76–81. 10.1016/j.jns.2011.03.039 21496826

[18] Sancho J Martínez D Bures E Díaz JL Ponz A Servera E. Bulbar impairment score and survival of stable amyotrophic lateral sclerosis patients after noninvasive ventilation initiation. ERJ Open Res. (2018) 4:00159-2017. 10.1183/23120541.00159-2017 29670892 PMC5900060

[19] Brusasco V Pellegrino R Rodarte JR. Vital capacities in acute and chronic airway obstruction: dependence on flow and volume histories. Eur Respir J. (1997) 10:1316–20. 10.1183/09031936.97.10061316 9192935

[20] Chhabra SK. Forced vital capacity, slow vital capacity, or inspiratory vital capacity: which is the best measure of vital capacity? J Asthma. (1998) 35:361–5. 10.3109/02770909809075669 9669830

[21] Fortis S Corazalla EO Wang Q Kim HJ. The difference between slow and forced vital capacity increases with increasing body mass index: a paradoxical difference in low and normal body mass indices. Respir Care. (2015) 60:113–8. 10.4187/respcare.03403 25316893

[22] Olson TP Wilson TA Johnson BD Hyatt RE. History dependence of vital capacity in constricted lungs. J Appl Physiol (1985). (2010) 109:121–5. 10.1152/japplphysiol.01365.2009 20413425 PMC2904196

[23] Rong P Jawdat O. A novel physiologic marker of bulbar motor involvement in amyotrophic lateral sclerosis: Jaw muscle synergy. Clin Neurophysiol. (2021) 132:94–103. 10.1016/j.clinph.2020.09.030 33259978

[24] Leite Neto L França MC Jr Chun RYS. Speech intelligibility in people with amyotrophic lateral sclerosis (ALS). CoDAS. (2021) 33:e20190214. 10.1590/2317-1782/20202019214 33533830

[25] Kühnlein P Gdynia HJ Sperfeld AD Lindner-Pfleghar B Ludolph AC Prosiegel M. Diagnosis and treatment of bulbar symptoms in amyotrophic lateral sclerosis. Nat Clin Pract Neurol. (2008) 4:366–74. 10.1038/ncpneuro0853 18560390

[26] Cave R Bloch S. The use of speech recognition technology by people living with amyotrophic lateral sclerosis: a scoping review. Disabil Rehabil Assist Technol. (2021) 1–13. 10.1080/17483107.2021.1974961 34511007

[27] Adamske D Heyduck A Weidenmüller M Göricke B Frank T Olthoff AA-O. Dysphagia in amyotrophic lateral sclerosis: quantification of bulbar motor dysfunction. J Oral Rehabil. (2021) 48:1044–9. 10.1111/joor.13220 34185922

