# Supplementary material for: Comparison of Slow and Forced Vital Capacity on Ability to Evaluate Respiratory Function in Bulbar-Involved Amyotrophic Lateral Sclerosis
Source: Front Neurol. 2022 Jun 28;13:938256. doi: 10.3389/fneur.2022.938256 (PMC9275792; doi:10.3389/fneur.2022.938256)
Supplement: Supplementary file 1 [file Table_1.DOCX]

Supplementary Material

# Supplementary Table

The clinical features of 5 patients who were unable to perform PFTs.

| No. | 1 | 2 | 3 | 4 | 5 |
| --- | --- | --- | --- | --- | --- |
| Sex | Female | Male | Female | Male | Female |
| Age, y | 46 | 48 | 67 | 43 | 67 |
| BMI, kg/m^2^ | 25.5 | 26.7 | 19.7 | 23.3 | 18.1 |
| Onset site | Bulbar | Bulbar | Bulbar | Bulbar | Bulbar |
| Main symptoms | Dysarthria | Dysarthria | Dysarthria | Dysarthria | Dysarthria |
| Age of onset, y | 45 | 47 | 63 | 43 | 67 |
| Diagnostic delay, months | 12 | 8 | 33 | 6 | 7 |
| KCSS | 4 | 3 | 4 | 3 | 1 |
| ALSFRS-R | 34 | 39 | 22 | 43 | 46 |
| B subscore | 7 | 11 | 7 | 11 | 10 |
| PO_2_, mmHg | 64.4 | 83.7 | 71 | 78 | 95 |
| PCO_2_, mmHg | 42.1 | 42.7 | 45 | 43 | 40 |
| SO_2_, % | 92.3 | 96.3 | 93 | 96 | 97 |

Abbreviations PFTs, pulmonary function tests; BMI, body mass index; KCSS, King’s College staging system; ALSFRS-R, ALS functional rating scale-revised; B sub-score, bulbar sub-score of ALSFRS-R; PO_2_, partial pressure of oxygen; PCO_2_, partial pressure of carbon dioxide; SO_2_, saturation of arterial blood oxygen.
